# Retraction: Hsa_circ_0000673 is down-regulated in gastric cancer and inhibits the proliferation and invasion of tumor cells by targeting miR-532-5p

**DOI:** 10.1042/BSR-2018-0538_RET

**Published:** 2022-07-14

**Keywords:** cell proliferation, gastric cancer, Hsa_circ_0000673, Invasion, miR-532-5p

This article is being retracted from *Bioscience Reports* at the request of the Editor-in-Chief and the Editorial Board following receipt of a notification from several readers, alerting the Editorial Board to duplications in Figures 3C and 6D, after which a third duplication in Figure 6D was identified. Readers also identified that several panels from Figures 6C and D also appeared in the group’s previous publication (Wei et al. LDLRAD2 overexpression predicts poor prognosis and promotes metastasis by activating Wnt/β-catenin/EMT signaling cascade in gastric cancer. *Aging (Albany NY).* 2019 Oct 24;11(20):8951-8968. doi: 10.18632/aging.102359).

The authors have contacted the Journal to correct the Article however, given they were unable to provide the original images and the extent of the issues raised, the Editorial Board stand by the decision to retract the article. The authors do not agree to the Retraction.

